# Integrated clinical characteristics and omics analysis identifies a ferroptosis and iron-metabolism-related lncRNA signature for predicting prognosis and therapeutic responses in ovarian cancer

**DOI:** 10.1186/s13048-022-00944-y

**Published:** 2022-01-20

**Authors:** Songwei Feng, Han Yin, Ke Zhang, Mei Shan, Xuan Ji, Shanhui Luo, Yang Shen

**Affiliations:** 1 Department of Obstetrics and Gynaecology, Zhongda Hospital, School of Medicine, Southeast University, Nanjing, 210009 China; 2 Department of Obstetrics and Gynaecology, Nanguan Hospital, Suqian, China; 3 Department of Gynaecology, the Second Affiliated Hospital of Soochow University, Soochow University, Suzhou, China

**Keywords:** ferroptosis, iron-metabolism, lncRNAs, Prognosis, Ovarian cancer

## Abstract

**Background:**

Ferroptosis and iron-metabolism are regulated by Long non-coding RNAs (lncRNAs) in ovarian cancer (OC). Therefore, a comprehensive analysis of ferroptosis and iron-metabolism related lncRNAs (FIRLs) in OC is crucial for proposing therapeutic strategies and survival prediction.

**Methods:**

In multi-omics data from OC patients, FIRLs were identified by calculating Pearson correlation coefficients with ferroptosis and iron-metabolism related genes (FIRGs). Cox-Lasso regression analysis was performed on the FIRLs to screen further the lncRNAs participating in FIRLs signature. In addition, all patients were divided into two robust risk subtypes using the FIRLs signature. Receiver operator characteristic (ROC) curve, Kaplan–Meier analysis, decision curve analysis (DCA), Cox regression analysis and calibration curve were used to confirm the clinical benefits of FIRLs signature. Meanwhile, two nomograms were constructed to facilitate clinical application. Moreover, the potential biological functions of the signature were investigated by genes function annotation. Finally, immune microenvironment, chemotherapeutic sensitivity, and the response of PARP inhibitors were compared in different risk groups using diversiform bioinformatics algorithms.

**Results:**

The raw data were randomized into a training set (*n* = 264) and a testing set (n = 110). According to Pearson coefficients between FIRGs and lncRNAs, 1075 FIRLs were screened for univariate Cox regression analysis, and then LASSO regression analysis was used to construct 8-FIRLs signature. It is worth mentioning that a variety of analytical methods indicated excellent predictive performance for overall survival (OS) of FIRLs signature (*p* < 0.05). The multivariate Cox regression analysis showed that FIRLs signature was an independent prognostic factor for OS (*p* < 0.05). Moreover, significant differences in the abundance of immune cells, immune-related pathways, and drug response were excavated in different risk subtypes (*p* < 0.05).

**Conclusion:**

The FIRLs signature can independently predict overall survival and therapeutic effect in OC patients.

**Supplementary Information:**

The online version contains supplementary material available at 10.1186/s13048-022-00944-y.

## Introduction

Globally, ovarian cancer (OC) is an important cause of gynaecological cancer-related death. Because a large proportion of patients lack specific clinical manifestations in the early stage, resulting in 70% of patients being diagnosed at an advanced stage [1]. Therefore, exploring new diagnostic strategies for OC patients is currently an urgent problem.

Iron is a trace element required by the human body, so its shortage or excess can have a variety of effects on biological processes [2]. Cancer cells rely more on iron for proliferation and are far more vulnerable to the iron deficiency than non-cancerous cells [3]. Particularly noteworthy is that high iron concentrations can cause cell death by membrane lipid peroxidation, termed ferroptosis [4]. Ferroptosis has also been discovered as a possible preventive or therapeutic strategy for cancer cell death, particularly in resistant cancers to traditional therapies [5]. Some investigations have discovered a possible role for ferroptosis and iron metabolism in OC progression [6–8], although the precise molecular mechanisms are yet unknown. In the meantime, lncRNAs are defined as non-protein-coding transcripts larger than 200 nucleotides [9]. LncRNAs have been shown to play major regulatory roles in various disease processes, including OC [10, 11]. LncRNAs have been shown to play significant regulatory roles in various disease processes, including OC [12, 13]. At present, there are many studies using lncRNAs expression to predict the prognosis of cancer patients, such as a risk score system based on co-expression network analysis [14], four prognosis-associated lncRNAs as biomarkers in OC [15], and lncRNAs-associated ceRNA network [16]. However, the clinical significance of most FIRLs has not been intensely studied in OC patients. Therefore, it is necessary to investigate the clinical value of lncRNAs related to iron metabolism and ferroptosis and screen out hub lncRNAs for predicting OS in OC patients.

In this study, we identified a FILRs signature based on 8-FIRLs (AC138904.1, AP005205.2, AC007114.1, LINC00665, UBXN10-AS1, AC083880.1, LINC01558, and AL023583.1) that showed an ability to distinguish OC patients into different risk groups, and clinical benefits in survival prediction were confirmed. In conclusion, FILRs signature played a significant role in OC patients and may be used as a predictive biomarker.

## Materials and methods

### Datasets and data pre-processing

The OC-clinical data, OC-RNA sequencing profiles, and normal ovarian epithelial tissue RNA sequencing profiles were obtained from The Cancer Genome Atlas (TCGA) [17] and GTEx database [18] using UCSC Xena. We excluded OC patients without RNA sequencing, survival time, or repeat sequencing, and finally, only 374 patients were retained for subsequent analysis. At a ratio of 3:7, the total OC patients were divided into two sets (training set and testing set) using the caret package in R software. Meanwhile, lncRNAs and protein-coding genes were identified based on annotation documents of the GENCODE database [19]. In addition, 296 FIRGs (**Table. S1**) were extracted based on previous studies [20], including ferroptosis regulators, ferroptosis markers, ferroptosis pathway, Iron uptake and transport, and Iron ion homeostasis, etc. It is worth mentioning that somatic mutation data were also obtained from the TCGA database, and homologous recombination repair (HRR) related genes were obtained from the previous reference [21].

## Construction of a signature and two nomograms based on FIRLs

Prognostic lncRNAs (*p*-value < 0.01) were screened using Cox regression, and LASSO regression analysis was used to identify FIRLs in risk signature. The risk score was calculated as follows:

$${\sum }_{\mathrm{i}=1}^{\mathrm{n}}{\mathrm{Coef}}_{\mathrm{i}}*{\mathrm{x}}_{\mathrm{i}}$$

where Coef is the coefficient of each gene, x is the expression value of each selected FIRL, and n is the number of FIRL. Based on the coefficients of the above formula, we use two packages (rms and replot) in R software to build Nomograms.

## Exploring clinical benefit of signature

According to the above formula, the risk score of each OC patient was calculated. PCA analysis, AUC and DCA curve assessed risk signature for the ability to predict survival. We calculated the risk score of each patient in the training cohort for determining the median value, which is used to select high-risk and low-risk groups. Moreover, Kaplan–Meier survival analysis suggested that the difference between different risk groups.

### Immune and functional enrichment analysis

In exploring differences in immune cell infiltration, we simultaneously used 6 algorithms (TIMER, CIBERSORT, QUANTISEQ, MCP-counter, XCELL, and EPIC) to estimate the abundances of immune cells in different risk groups distinguished by FIRLs signature. Moreover, we used the ssGSEA algorithm to quantify immune functions and pathways. More importantly, we also explored immune checkpoint-related gene expression levels in different risk groups. Finally, GO and KEGG functional enrichment analysis of FIRLs signature was conducted.

### Drug sensitivity analysis

The IC50 was calculated using pRRophetic package in R software, and the chemotherapeutic medications were obtained from the Genomics of Drug Sensitivity in Cancer (GDSC) database [22].

### Comparison of survival prediction value of different signatures

To highlight the substantial prognostic value of FIRLs signatures, we compared the efficacy of other signatures from different references. Zhang et al. identified a glycolysis-related gene signature for OC patients, including ISG20, CITED2, PYGB,

IRS2, ANGPTL4, TGFBI, LHX9, PC, and DDIT4 [23]. Zhou et al. identified a DNA methylation-driven genes signature, including PON3, MFAP4, AKAP12, and BHMT2 [24]. Moreover, Zheng et al. developed a risk stratification system based on glycolysis-related lncRNAs, including AC133644.2, CTD-2396E7.11, CTD-3065, J16.9, LINC00240, and TMEM254-AS1 [25]. In 374 patients with OC, we performed Lasso-Cox analysis on the above genes to calculate the corresponding risk score. Finally, C-index was used to compare the predictive ability of the different models.

#### Statistical analysis

All statistical analyses were performed using the R software (v.4.0.1). Detailed statistical methods for transcriptome data processing are covered in the above section. *P* < 0.05 was considered statistically significant.

## Result

Identification of FIRLs.

**Fig. 1:**
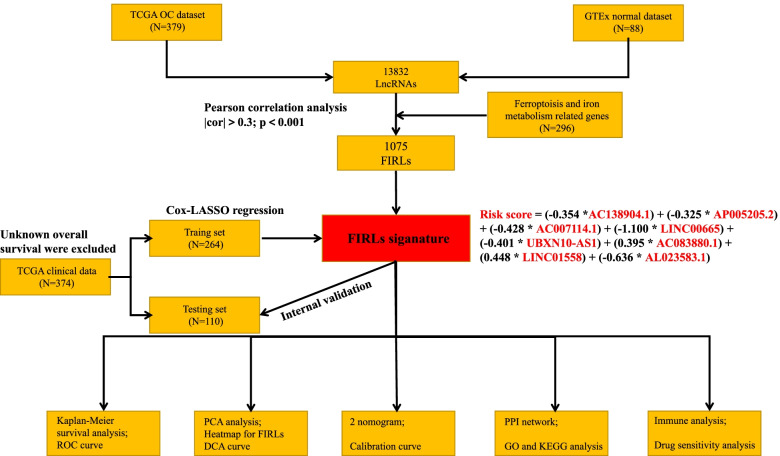
A flowchart of the study

Pearson correlation analysis was performed on the identified 13,832 lncRNAs and 296 FIRGs. Ultimately, we screened out 1075 FIRLs for subsequently bioinformatic analysis. It is worth mentioning that construction and validation for FIRLs signature were carried out according to the flowchart, as shown in Fig. 1. Taken together, our data showed that 1075 FIRLs was identified in OC samples.

### Derivation of a FIRLs signature in OC patients

**Fig. 2:**
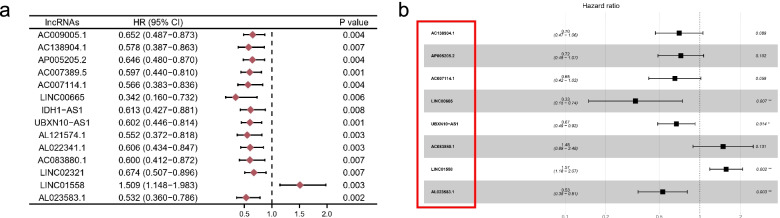
Derivation of FIRLs signature for predicting overall survival. **a** The result of univariate cox regression analysis in 1075 FIRLs; **b** A forest plot of 8 FIRLs participating in signature construction

We randomly divided 374 OC patients into a testing set (110 patients) and a training set (274 patients) to construct and validate the signature. Subsequently, 14 lncRNAs (*p* < 0.01) were significantly correlated with the survival by univariate Cox regression analysis in the training set, as shown in Fig. 2a. We aimed to avoid the occurrence of collinearity of transcriptome data, and LASSO regression analysis was used to screen out further 8-lncRNAs, which constituted a prognostic risk signature of FIRLs (Table.1, Fig. 2b). Finally, combining the expression of 8-FIRLs and regression coefficients in multivariate Cox regression analysis, the risk score of OC patients is calculated as follows: Risk score = (-0.354 *AC138904.1) + (-0.325 * AP005205.2) + (-0.428 * AC007114.1) + (-1.100 * LINC00665) + (-0.401 * UBXN10-AS1) + (0.395 * AC083880.1) + (0.448 * LINC01558) + (-0.636 * AL023583.1). Taken together, our data showed that an 8-FIRLs signature was derived in the training set.

**Table 1:** Multivariate Cox regression analysis

LncRNA	coef	HR	HR.95L	HR.95H	P-value
AC138904.1	-0.354	0.702	0.467	1.055	0.089
AP005205.2	-0.325	0.722	0.489	1.067	0.102
AC007114.1	-0.428	0.652	0.418	1.017	0.059
LINC00665	-1.100	0.333	0.150	0.740	0.007
UBXN10-AS1	-0.401	0.669	0.486	0.922	0.014
AC083880.1	0.395	1.485	0.889	2.479	0.131
LINC01558	0.448	1.565	1.185	2.068	0.002
AL023583.1	-0.636	0.529	0.348	0.806	0.003

### Clinical benefit of FIRLs signature

**Fig. 3:**
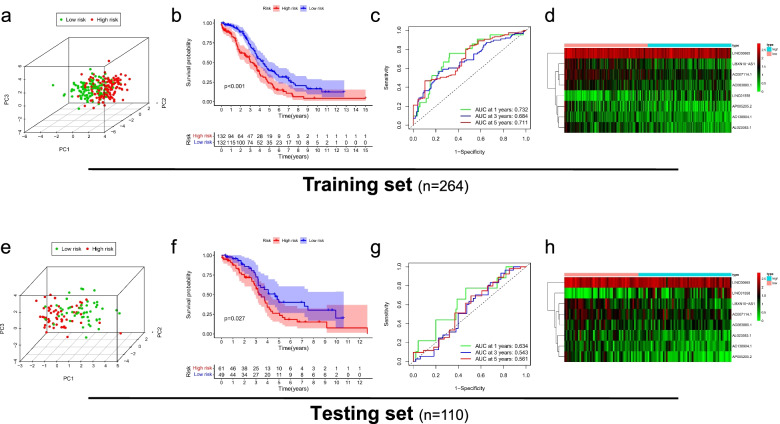
Clinical benefit of FIRLs signature for patients with OC. **a** PCA analysis of high-risk and low-risk groups in the the training set; **b** Kaplan–Meier survival analysis of high-risk and low-risk groups in the training set; **c** ROC curve of 1,3,5 year survival prediction in the training set; **d** A heatmap of 8 FIRLs participating in signature construction in the training set; **e** PCA analysis of high-risk and low-risk groups in the testing set; **f** Kaplan–Meier survival analysis of high-risk and low-risk groups in the testing set; **g** ROC curve of 1,3,5 year survival prediction in the testing set; **h** A heatmap of 8 FIRLs participating in signature construction in the testing set

**Fig. 4:**
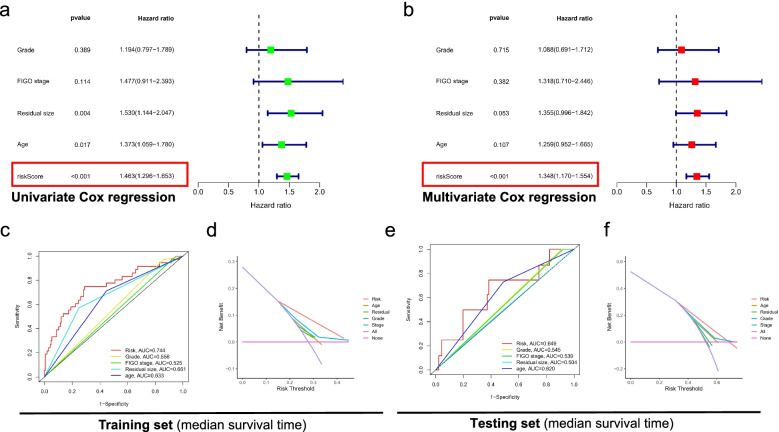
Cox regression analysis and DCA analysis of FIRLs signature for patients with OC. **a** Forest plot of univariate Cox regression analysis in all patients; **b** Forest plot of multivariate Cox regression analysis in all patients; **c** ROC curve of clinicopathological features including FIRLs signature in the training set; **d** DCA analysis of clinicopathological features including FIRLs signature in the training set; **e** ROC curve of clinicopathological features including FIRLs signature in the testing set; **f** DCA analysis of clinicopathological features including FIRLs signature in the testing set

We calculated the risk score of OC patients in the testing set and training set according to the above formula. According to the median value in the training set, the population in each group was divided into a high-risk group and a low-risk group. Firstly, the PCA analysis confirmed a risk signature's classification ability in the testing set and training set, as shown in Fig. 3a, e. Subsequently, Kaplan–Meier survival analysis showed that OS of the high-risk group was significantly shorter than that of the low-risk group (training set: *p* < 0.05, as shown in Fig. 3b; testing set: *p* < 0.05, as shown in Fig. 3f), which indicates that FIRLs signature has an excellent predictive value. We evaluated the predictive sensitivity and specificity of FIRLs signature by ROC curve. The AUC of the training set and the testing set at 1, 3, and 5 years reached 0.732, 0.684, 0.711 and 0.634, 0.577, 0.525, respectively, as shown in Fig. 3c, g. In addition, the heatmap of 8 FIRLs expressions in the high- and low-risk groups is shown in Fig. 3d, h. Finally, we performed univariate and multivariate Cox regression analysis of FIRLs signature and clinical characteristics in total patients. The results showed that FIRLs signature is an independent prognostic factor for OC patients (*p* < 0.001), as shown in Fig. 4a, b. What is exciting is that the DCA curve and ROC curve showed that FIRLs signature to predict the median survival time is significantly better than traditional clinical characteristics in testing set and training set, as shown in Fig. 4c-f. Taken together, our data showed that FIRLs signature has a superior clinical benefit for OC patients.

### Construction of visual model

**Fig. 5:**
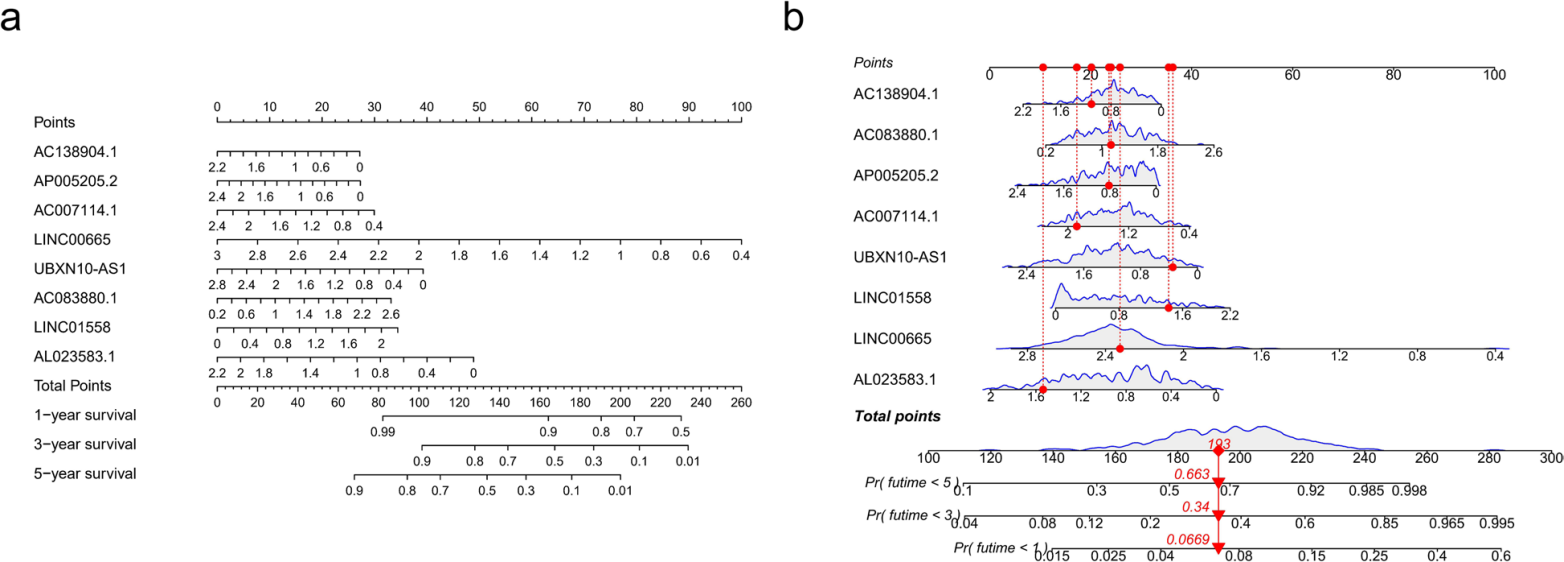
Construction of visual model. **a** A nomogram plotted by rms package for predicting the OS at 1, 3, and 5 years; **b** A nomogram plotted by regplot package for predicting the OS at 1, 3, and 5 years

Considering that the formula of the FIRLs signature is complicated in routine clinical work, the nomogram [26] can intuitively apply to clinical work, so we visualized the risk signature based on the above risk formula. As shown in Fig. 5a-b, we plotted two nomograms based on the same risk formula. Moreover, the calibration curve of the nomogram showed that the prediction curves are close to the standard curve in the testing set and training set, which indicates that the predicted survival rate is closely related to the actual rates at 1, 3, and 5 years, as shown in Fig. S1. Taken together, our data showed that our nomograms could intuitively apply to clinical work.

**Correlation of the risk score** with clinicopathological features.

**Fig. 6:**
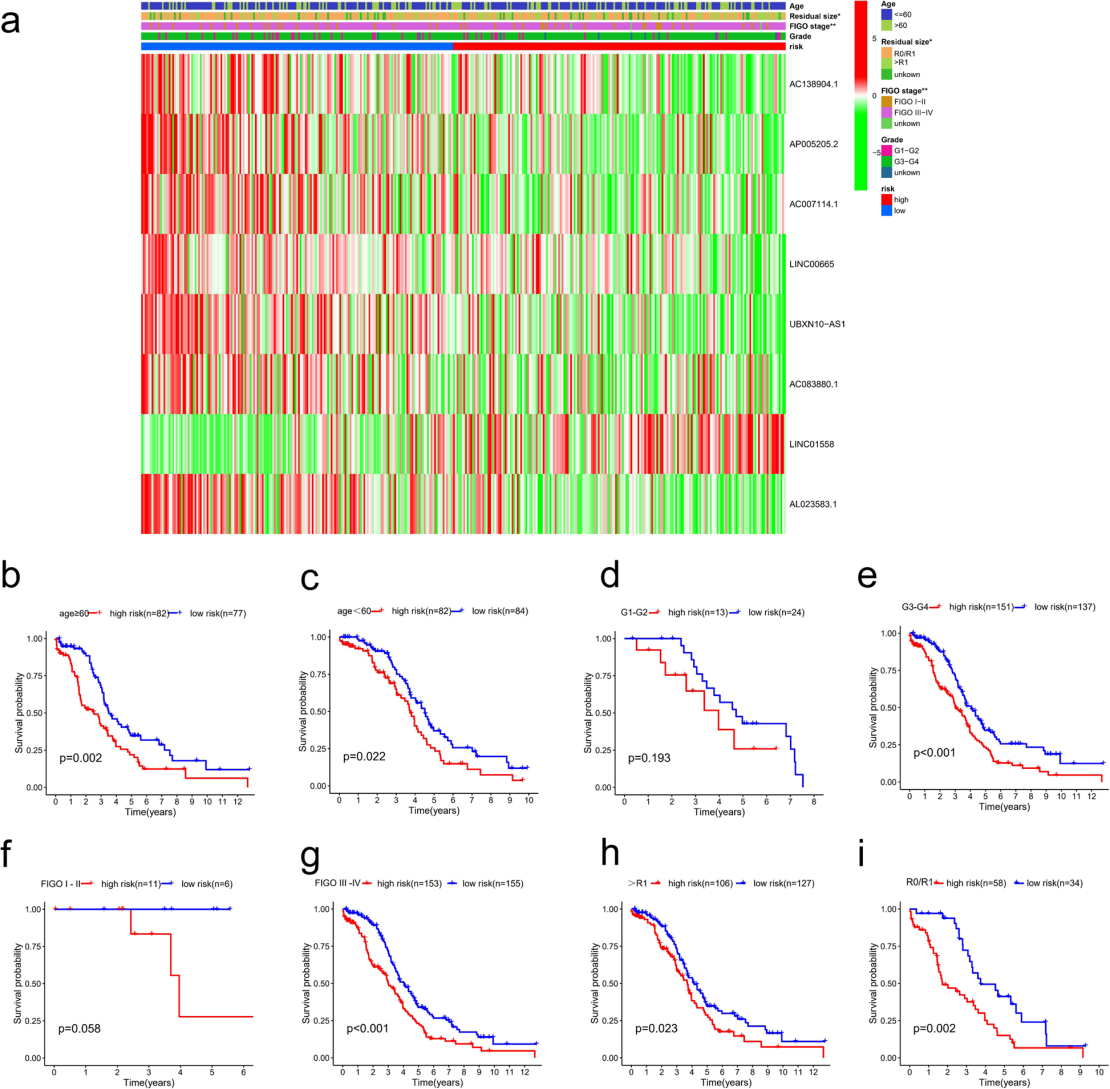
Correlation of the risk score of with clinicopathological features. **a** 8 FIRLs expression in all patients and correlation between FIRLs signature and clinicopathological features; **b**, **c** The survival differences between different risk groups stratified by age; **d**, **e** The survival differences between different risk groups stratified by grade; **f**, **g** The survival differences between different risk groups stratified by FIGO stage; **h**, **i** The survival differences between different risk groups stratified by residual tumor size; *P < 0.05; **P < 0.01; ***P < 0.001

We used the chi-square test to study whether the high- and low-risk groups based on FIRLs signature is involved in the development of OC. Our heatmap showed that there were significant differences between the high-risk group and the low-risk group in FIGO staging (*P* < 0.01) and residual tumour size (*P* < 0.05), as shown in Fig. 6a. To further explore the predictive efficiency of FIRLs signature in different clinical characteristics (Fig. 6b-i), the following clinical variables were used for analysis: age (≤ 60 and > 60), FIGO stage (I—II, and III—IV), pathological grade (G1- 2 and G 3–4), residual tumour size (R0/R1 and > R1). In the remaining subgroups except for the FIGO I-II (p = 0.058) and G1-G2 subgroups (p = 0.193), the results revealed that FIRLs signature has prognostic significance between different risk groups. Particularly worth mentioning is that the OS of patients in the high-risk group was significantly lower than that of the low-risk patients in most subgroups (*P* < 0.05). Taken together, our findings revealed that the FIRLs signature plays a pivotal role in predicting the prognosis in patients with OC.

### Functional enrichment about FIRLs signature

**Fig. 7:**
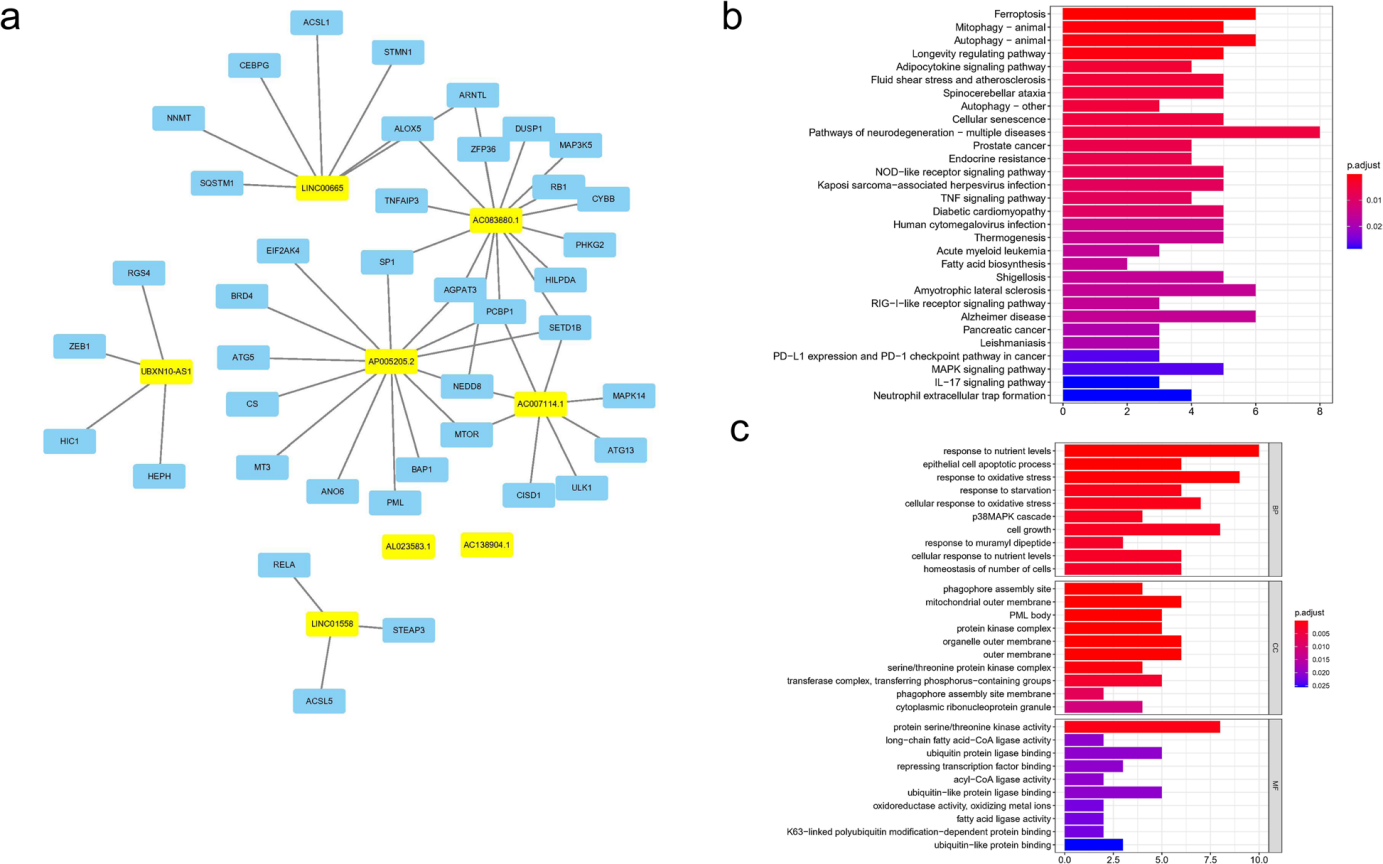
Functional enrichment analysis based on FIRLs signature. **a** Construction of PPI network including 8 LncRNAs and 40 proteins en-coding genes co-expressed; **b** KEGG enrichment analysis; **c** GO enrichment analysis

We screened out the 40 FIRGs co-expressed with 8-LncRNAs, as shown in Fig. 7a. KEGG enrichment analysis showed that related mRNAs were enriched in ferroptosis, mitophagy, and autophagy pathways, etc., as shown in Fig. 7b. GO enrichment analysis showed that 40 mRNAs were mainly related to response to nutrient levels and epithelial cell apoptotic process in BP section, mitochondrial outer membrane and protein kinase complex in CC section, and protein serine/threonine kinase activity and long-chain fatty acid-CoA ligase activity in MF section (Fig. 7c). In addition, we explored the expression of 8 lncRNAs in clinical samples. As expected, most of the lncRNAs (7/8) in OC samples were up-regulated except for AP005205.2, as shown in Fig. S2. Taken together, our data showed that GO and KEGG analysis verified the relationship between FIRLs signature and iron metabolism from another perspective.

### Immune analysis based on FIRLs signature

**Fig. 8:**
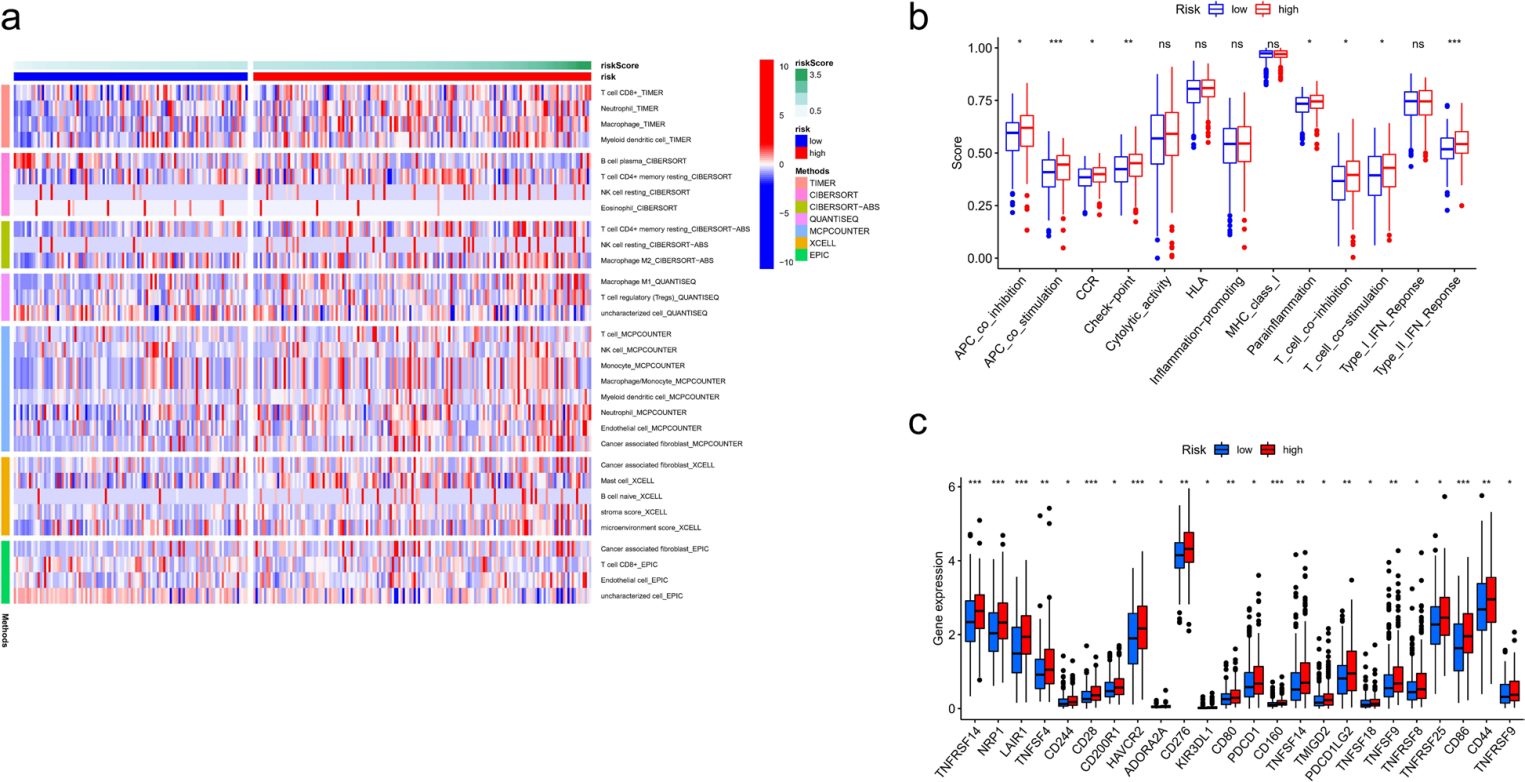
Immune analysis based on FIRLs signature. **a** A heatmap for different immune cells based on 6 algorithms; **b** Immune functions scores in different groups; **c** Expression of immune checkpoints in different groups.

To comprehensively explore the relationship between different risk groups and immune cell infiltration, we plotted the heatmap of immune infiltration based on 6 algorithms. Specific immune cells differed significantly among risk subgroups, such as Macrophages, T cells, NK cell resting, etc. (Fig. 8a). Interestingly, analysis of immunologic function confirmed significant differences between low- and high-risk groups for other immunological functions except for cytolytic activity, HLA, inflammation-promoting, MHC class I, and Type_I_IFN response (*P* > 0.05), as shown in Fig. 8b. Meanwhile, the boxplot showed immune checkpoints mRNA were up-regulated in the high-risk group compared to the low-risk group, as shown in Fig. 8c. Taken together, our data showed that FIRLs signature was correlated with immune cell infiltration and immunotherapy to a certain extent.

### Drug effectiveness analysis based on FIRLs signature

**Fig. 9:**
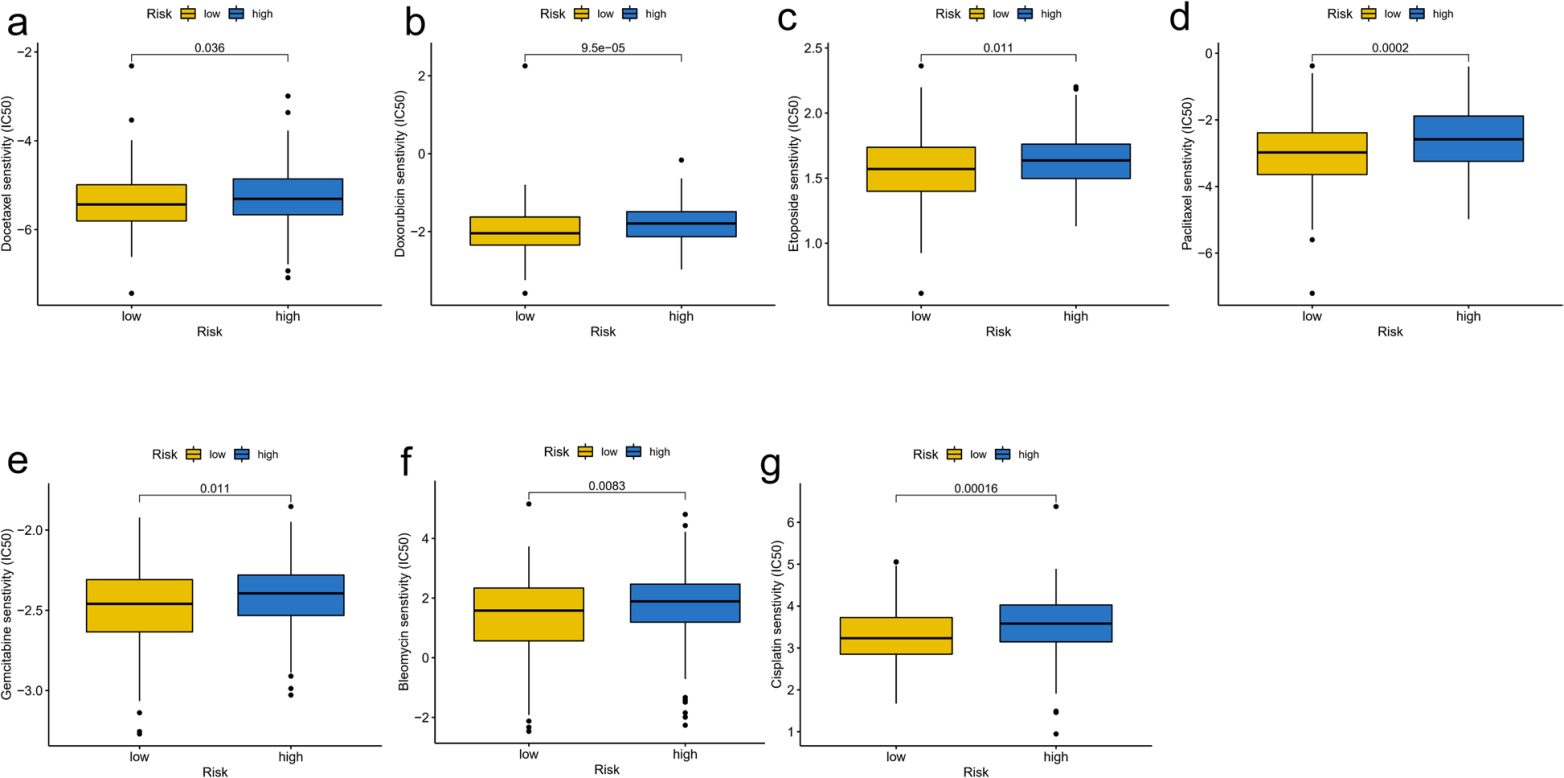
Drug sensitivity analysis in different risk groups based on FIRLs signature. **a** IC50 levels of Docetaxel; **b** IC50 levels of Doxorubicin; **c** IC50 levels of Etoposide; **d** IC50 levels of Paclitaxel; **e** IC50 levels of Gemcitabine; **f** IC50 levels of Bleomycin; **g** IC50 levels of Cisplatin

**Fig. 10:**
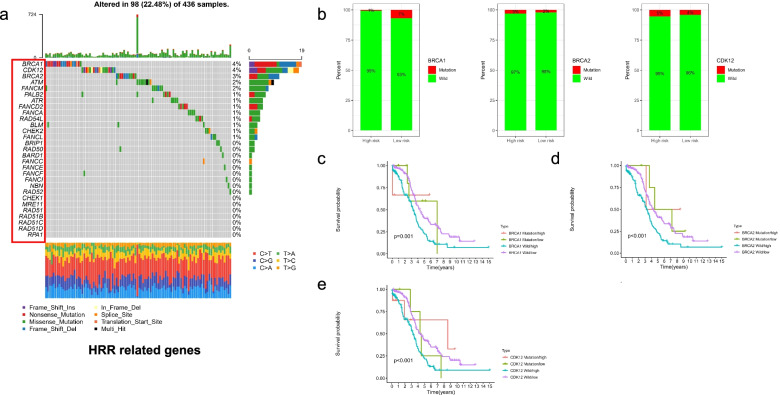
Analysis of homologous recombination repair in different risk subgroups. **a** Landscape of HRR-related genes about mutation frequency; **b** Differences in the distribution of BRCA1, BRCA2 and CDK12 mutations among different groups; **c** Survival analysis of BRCA1 mutation and risk combination; **d** Survival analysis of BRCA2 mutation and risk combination; **e** Survival analysis of CDK12 mutation and risk combination

We investigated the drug sensitivity of chemotherapeutic agents often used in clinics in different risk subgroups. The IC50 values of 7 chemotherapeutic medicines were quantified in OC patients, and 5 were statistically different between risk groups. In detail, the IC50 levels of Docetaxel (Fig. 9a), Doxorubicin (Fig. 9b), Etoposide (Fig. 9c), Paclitaxel (Fig. 9d) and Gemcitabine (Fig. 9e), Bleomycin (Fig. 9f), and Cisplatin (Fig. 9g) were significantly higher in high-risk group (*P* < 0.05). It indicated that the OC patients in the low-risk group distinguished by FIRLs signature were more sensitive to the above chemotherapeutics. In addition, considering the critical correlation of HRR-related genes in OC patients for the maintenance therapy, we further analyzed the association of different risk groups with HRR-related genes. We found that BRCA1, BRCA2 and CDK12 were the top three genes (Fig. 10a). Mutations in BRCA1 and CDK12 were not statistically significant in TCGA-cohort; however, there was a statistically significant difference in BRCA1 gene mutation (Fig. 10b). Specifically, the low-risk group had a higher frequency of BRCA1 mutations. Meanwhile, we combined risk groups with mutations in BRCA1, BRCA2 and CDK12 for survival analysis. The results showed a statistically significant difference between the four groups, as shown in Fig. 10c-e. Taken together, our data showed that 7 chemotherapeutic agents and PARP inhibitors might have a better effect on patients in the low-risk group.

### Comparison of predictive value of 8-FIRLs with other risk signatures

**Fig. 11:**
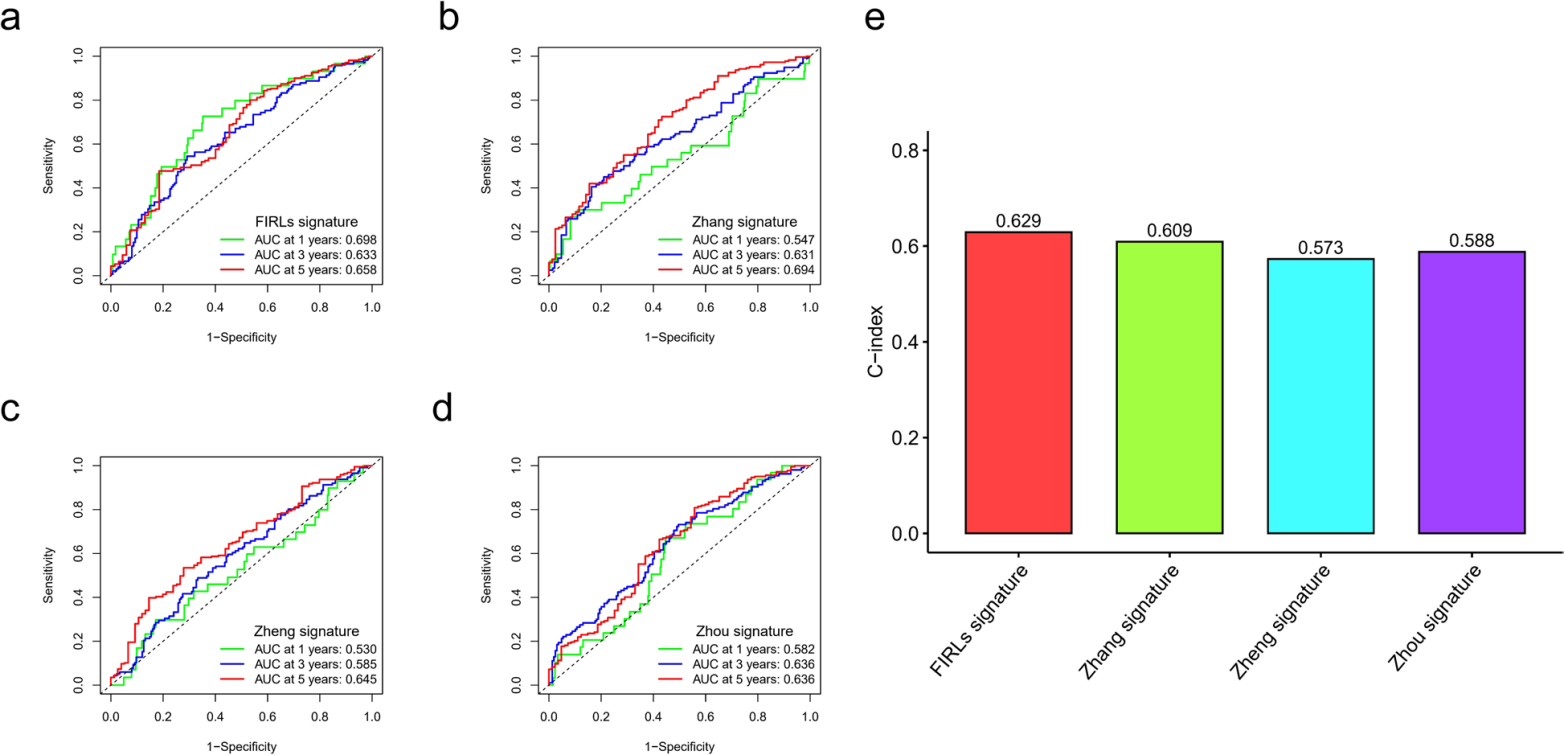
Comparison of predictive effectiveness in different risk signatures. **a** ROC analysis of FIRLs signature in TCGA cohort.; **b** ROC analysis of Zhang signature in TCGA cohort.; **c** ROC analysis of Zheng signature in TCGA cohort; **d** ROC analysis of Zhou signature in TCGA cohort; **e** C-index of different signatures

To highlight the prognostic value of FIRL signatures, we compared the efficacy of other signatures from different references. Zhang et al. identified a glycolysis-related gene signature for OC patients, and Zhou et al. identified a DNA methylation-driven genes signature. Moreover, Zheng et al. developed a risk stratification system based on glycolysis-related lncRNAs. In 374 patients with OC, we performed Lasso-Cox analysis on the above signatures to calculate the corresponding risk score and showed ROC analysis of 1, 3, and 5 years (Fig. 11a-d). The C-index value showed that the 8-FIRLs signature had the most robust predictive performance (Fig. 11e). However, it should not be ignored that other risk signatures can also stratify the risk of OC patients.

## Discussion

Excessive intracellular iron accumulation is caused by disturbances in iron metabolism, which can lead to ferroptosis [27]. In recent years, ferroptosis and iron metabolism have been reported to be crucially in multiple cancer, so the non-coding RNAs, especially lncRNAs, which regulate these two processes, have been extensively studied. Although currently Chen [28], Fei [29] and He [30] have constructed risk signature for survival prediction in OC patients based on autophagy-related genes and RNA-binding protein genes, respectively. However, to our knowledge, this is the first research in which prognostic FIRLs in OC patients have been identified and comprehensively analyzed. The signature is based on 8 FIRLs (AC138904.1, AP005205.2, AC007114.1, LINC00665, UBXN10-AS1 AC083880.1, LINC01558, and AL023583.1), can be used to guide prognostic and treatment decisions. Meanwhile, we also present two nomograms for visualizing the FIRLs signature.

lncRNAs has been proved to play an important role in the occurrence and development of tumors by bioinformatics methods or experiments. For example, Chen and his colleagues developed some advanced computational models that can be used effectively to identify disease-associated LncRNAs on a large scale [31]. Moreover, a a multi-label fusion collaborative matrix factorization (MLFCMF) approach was proposed for predicting lncRNA-disease associations, especially, their method finally obtains an AUC value of 0.8612 [32]. In addition to the above bioinformatics studies, the role of most lncRNAs in OC cell lines has also been explored, such as MSC-AS1 [33], TONSL-AS1 [34], and SNHG20 [35], etc. However, lncRNAs participating in risk signature have not been well explored in OC, and this suggests that our study is an indicator for Vivo and Vitro assays in the future.

In the development of OC, immune regulation is critical [36]. The quantity and proportion of immune cells invading a tumour are essential variables influencing cancer development and immunotherapy response [37] and being linked to patient prognosis. According to most reviews of tumour immunoediting theory [38], tumour cells with low-immunogenicity are often selected by the host to escape the anti-tumour immune response. This might lead to a rise in immunosuppressive cells and a decrease in immunoreactive cells. As a result, we anticipated that patients in different risk categories based FIRLs signatures would respond differently to immunotherapy. Among the essential immune cells, we found statistical difference infiltration of B cell plasma, T cell CD4 + memory resting, NK cell resting, etc. The above findings suggest that the poorer prognosis of high-risk patients is due to higher immunosuppression in the tumour microenvironment, and these differences contribute to tumour progression. In addition, Immunotherapies based on checkpoint inhibitors have improved the survival of patients with OC [39]. Our results suggest significant differences in the expression of immune checkpoint related genes in different groups, indicating differences in immunotherapy sensitivity. Meanwhile, we further explored the differences in chemotherapy drug sensitivity between the two risk groups. Our results showed that the IC50 levels of multiple chemotherapy drugs were significantly higher in a high-risk group, indicating that the OC patients in the low-risk group were more sensitive to these drugs.

However, there are numerous limitations to our study that should be considered. To begin, our research was only based on the TCGA database. When extending our findings to patients of different ethnicities, caution is advised. Second, the FIRLs signature must be validated in multicenter cohorts in the future. Finally, more functional experiments will be necessary to confirm our findings and better understand the roles of 8- FIRLs in OC.

## Conclusions

In summary, a novel FIRLs signature consisting of 8 lncRNAs was identified for OC patients. Besides, the signature may help guide individual therapy and improve patients’ prognoses for OC. Since studies on the mechanism and relationships among these FIRLs in OC are still rare, further investigation in depth is warranted to validate the clinical application value and uncover the underlying pathways.

## Supplementary Information

**Additional file 1.** Table S1. A list of FIRGs

**Additional file 2.** Figure S1. Calibration curve of nomogram. **(a-b)** Calibration curve of nomogram based on FIRLs signature for OS prediction at 1 year **(a)**, 3 year **(b)** , and 5 year **(c)** in the training set; **(d-f)** Calibration curve of nomogram based on FIRLs signature for OS prediction at 1 year **(d)**, 3 year **(e)**, and 5 year **(f)** in the testing set.

**Additional file 3.** Figure S2. The expression of 8-FIRLs in normal and OC samples. **(a)** The expression of AC138904.1; **(b)**The expression of AP005205.2; **(c)** The expression of AC007114.1; **(d)** The expression of LINC00665; **(e)** The expression of UBXN10-AS1; **(f)** The expression of AC083880.1; **(g)** The expression of LINC01558; **(h)** The expression of AL023583.1; ^*^P < 0.05; ^**^P < 0.01; ^***^P < 0.001.

## Data Availability

The following information was supplied regarding data availability: TCGA datasets were downloaded from UCSC Xena (https://xenabrowser.net/).

## Abbreviations

OC: Ovarian cancer
lncRNAs: Long non-coding RNAs
FIRLs: Ferroptosis and iron-metabolism related lncRNAs
FIRGs: Ferroptosis and iron-metabolism related genes
ROC: Receiver operator characteristic
K-M: Kaplan–Meier
DCA: Decision curve analysis
OS: Overall survival
