# Supplementary material for: Integrated clinical characteristics and omics analysis identifies a ferroptosis and iron-metabolism-related lncRNA signature for predicting prognosis and therapeutic responses in ovarian cancer
Source: J Ovarian Res. 2022 Jan 20;15:10. doi: 10.1186/s13048-022-00944-y (PMC8772079; doi:10.1186/s13048-022-00944-y)
Supplement: Supplementary file 1 — Additional file 1. Table S1. A list of FIRGs [file 13048_2022_944_MOESM1_ESM.docx]

**Table S1. A list of FIRGs.**

| **Ferroptosis and iron-metabolism related genes** | **Name** |
| --- | --- |
| PRNP | prion protein |
| VDAC3 | voltage dependent anion channel 3 |
| CYBB | cytochrome b-245 beta chain |
| PCBP1 | poly(rC) binding protein 1 |
| PCBP2 | poly(rC) binding protein 2 |
| ACSL1 | acyl-CoA synthetase long chain family member 1 |
| ACSL5 | acyl-CoA synthetase long chain family member 5 |
| ACSL6 | acyl-CoA synthetase long chain family member 6 |
| ACSF2 | Acyl-CoA synthetase family member 2 |
| GCLM | glutamate-cysteine ligase modifier subunit |
| GSS | glutathione synthetase |
| MAP1LC3B | microtubule associated protein 1 light chain 3 beta |
| MAP1LC3C | microtubule associated protein 1 light chain 3 gamma |
| SAT2 | spermidine/spermine N1-acetyltransferase family member 2 |
| SLC11A2 | solute carrier family 11 member 2 |
| SLC39A14 | solute carrier family 39 member 14 |
| SLC39A8 | solute carrier family 39 member 8 |
| ALOX12 | Arachidonate 12-lipoxygenase, 12S type |
| ALOX15 | Arachidonate 15-lipoxygenase |
| CS | Citrate synthase |
| EMC2 | ER membrane protein complex subunit 2 |
| ALOX5 | Arachidonate 5-lipoxygenase |
| NOX1 | Nicotinamide adenine dinucleotide phosphate (NADPH) oxidase (NOX) 1 |
| CYBB | Cytochrome b-245 beta chain |
| NOX3 | Nicotinamide adenine dinucleotide phosphate (NADPH) oxidase (NOX) 3 |
| NOX4 | Nicotinamide adenine dinucleotide phosphate (NADPH) oxidase (NOX) 4 |
| NOX5 | Nicotinamide adenine dinucleotide phosphate (NADPH) oxidase (NOX) 5 |
| DUOX1 | Dual oxidase 1 |
| DUOX2 | Dual oxidase 2 |
| G6PD | Glucose-6-phosphate dehydrogenase |
| PGD | Phosphoglycerate dehydrogenase |
| VDAC2 | Valtage-dependent anion channels 2 |
| PIK3CA | Phosphatidylinositol-4,5-bisphosphate 3-kinase catalytic subunit alpha |
| FLT3 | Fms related tyrosine kinase 3 |
| SCP2 | Sterol carrier protein 2 |
| TP53 | Tumor protein p53 |
| LPCAT3 | Lysophosphatidylcholine acyltransferase 3 |
| NRAS | NRAS proto-oncogene, GTPase |
| KRAS | KRAS proto-oncogene, GTPase |
| HRAS | HRas proto-oncogene, GTPase |
| TFR2 | Transferrin receptor 2 |
| SLC38A1 | Solute carrier family 38 member 1 |
| SLC1A5 | Solute carrier family 1 member 5 |
| CARS1 | Cysteinyl-tRNA synthetase 1 |
| KEAP1 | Kelch like ECH associated protein 1 |
| ATF3 | Activating transcription factor 3 |
| ATG5 | Autophagy related 5 |
| ATG7 | Autophagy related 7 |
| NCOA4 | Nuclear receptor coactivator 4 |
| ATF4 | Activating transcription factor 4 |
| ALOX12B | Arachidonate 12-lipoxygenase, 12R type |
| ALOX15B | Arachidonate 15-lipoxygenase type B |
| ALOXE3 | Arachidonate lipoxygenase 3 |
| PHKG2 | Phosphorylase kinase catalytic subunit gamma 2 |
| ACO1 | Aconitase 1 |
| GLS2 | Glutaminase 2 |
| ULK1 | Unc-51 like autophagy activating kinase 1 |
| ATG3 | Autophagy related 3 |
| ATG4D | Autophagy related 4D cysteine peptidase |
| BECN1 | Beclin 1 |
| MAP1LC3A | Microtubule associated protein 1 light chain 3 alpha |
| GABARAPL2 | GABA type A receptor associated protein like 2 |
| GABARAPL1 | GABA type A receptor associated protein like 1 |
| ATG16L1 | Autophagy related 16 like 1 |
| WIPI1 | WD repeat domain, phosphoinositide interacting 1 |
| WIPI2 | WD repeat domain, phosphoinositide interacting 2 |
| SNX4 | Sorting nexin 4 |
| ATG13 | Autophagy related 13 |
| ULK2 | Unc-51 like autophagy activating kinase 2 |
| SAT1 | Spermidine/spermine N1-acetyltransferase 1 |
| EGFR | Epidermal growth factor receptor |
| MAPK3 | Mitogen-activated protein kinase 3 |
| MAPK1 | Mitogen-activated protein kinase 1 |
| BID | BH3 interacting domain death agonist |
| ZEB1 | Zinc finger E-box binding homeobox 1 |
| DPP4 | Dipeptidyl peptidase 4 |
| CDKN2A | Cyclin dependent kinase inhibitor 2A |
| PEBP1 | Phosphatidylethanolamine binding protein 1 |
| SOCS1 | Suppressor of cytokine signaling 1 |
| CDO1 | Cysteine dioxygenase type 1 |
| MYB | MYB proto-oncogene, transcription factor |
| MAPK8 | Mitogen-activated protein kinase 8 |
| MAPK9 | Mitogen-activated protein kinase 9 |
| ATP5MC3 | ATP synthase membrane subunit c locus 3 |
| GOT1 | Glutamic-oxaloacetic transaminase 1 |
| PRKAA2 | Protein kinase AMP-activated catalytic subunit alpha 2 |
| PRKAA1 | Protein kinase AMP-activated catalytic subunit alpha 1 |
| BAP1 | BRCA1 associated protein 1 |
| ABCC1 | ATP binding cassette subfamily C member 1 |
| MIR6852 | microRNA 6852 |
| ACVR1B | Activin A receptor type 1B |
| TGFBR1 | Transforming growth factor beta receptor 1 |
| EPAS1 | Endothelial PAS domain protein 1 |
| HILPDA | Hypoxia inducible lipid droplet associated |
| IFNG | Interferon gamma |
| ANO6 | Anoctamin 6 |
| LPIN1 | Lipin 1 |
| CBS | Cystathionine beta-synthase |
| TNFAIP3 | TNF alpha induced protein 3 |
| TLR4 | Toll like receptor 4 |
| ATM | ATM serine/threonine kinase |
| YY1AP1 | YY1 associated protein 1 |
| EGLN2 | Egl-9 family hypoxia inducible factor 2 |
| MIOX | Myo-inositol oxygenase |
| TAZ | Tafazzin |
| MTDH | Metadherin |
| IDH1 | Isocitrate dehydrogenase (NADP(+)) 1 |
| SIRT1 | Sirtuin 1 |
| FBXW7 | F-box and WD repeat domain containing 7 |
| PANX1 | Pannexin 1 |
| DNAJB6 | DnaJ heat shock protein family (Hsp40) member B6 |
| BACH1 | BTB domain and CNC homolog 1 |
| ACSL4 | Acyl-CoA synthetase long chain family member 4 |
| LONP1 | Lon peptidase 1, mitochondrial |
| CHAC1 | ChaC glutathione specific gamma-glutamylcyclotransferase 1 |
| AKR1C2 | Aldo-keto reductase family 1 member C2 |
| AKR1C3 | Aldo-keto reductase family 1 member C3 |
| RB1 | RB transcriptional corepressor 1 |
| CP | ceruloplasmin |
| HSF1 | Heat shock transcription factor 1 |
| GCLC | Glutamate-cysteine ligase catalytic subunit |
| SQSTM1 | Sequestosome 1 |
| NQO1 | NAD(P)H quinone dehydrogenase 1 |
| EIF2S1 | Eukaryotic translation initiation factor 2 subunit 1 |
| MUC1 | Mucin 1, cell surface associated |
| MT1G | Metallothionein 1G |
| CISD1 | CDGSH iron sulfur domain 1 |
| FANCD2 | FA complementation group D2 |
| ELAVL1 | ELAV like RNA binding protein 1 |
| HSPA5 | Heat shock protein family A (Hsp70) member 5 |
| HELLS | Helicase, lymphoid specific |
| SCD | Stearoyl-CoA desaturase |
| FADS2 | Fatty acid desaturase 2 |
| SRC | SRC proto-oncogene, non-receptor tyrosine kinase |
| STAT3 | Signal transducer and activator of transcription 3 |
| PML | Promyelocytic leukemia |
| MTOR | Mechanistic target of rapamycin kinase |
| NFS1 | NFS1 cysteine desulfurase |
| TP63 | Tumor protein p63 |
| CDKN1A | Cyclin dependent kinase inhibitor 1A |
| MIR137 | microRNA 137 |
| ENPP2 | Ectonucleotide pyrophosphatase/phosphodiesterase 2 |
| FH | Fumarate hydratase |
| CISD2 | CDGSH iron sulfur domain 2 |
| MIR9-1 | microRNA 9-1 |
| MIR9-2 | microRNA 9-2 |
| MIR9-3 | microRNA 9-3 |
| FTH1 | Ferritin heavy chain 1 |
| ISCU | Iron-sulfur cluster assembly enzyme |
| ACSL3 | Acyl-CoA synthetase long chain family member 3 |
| OTUB1 | OTU deubiquitinase, ubiquitin aldehyde binding 1 |
| CD44 | CD44 molecule (Indian blood group) |
| BRD4 | Bromodomain containing 4 |
| PRDX6 | Peroxiredoxin 6 |
| MIR17 | microRNA 17 |
| NF2 | Neurofibromin 2 |
| ARNTL | Aryl hydrocarbon receptor nuclear translocator like |
| HIF1A | Hypoxia inducible factor 1 subunit alpha |
| JUN | Jun proto-oncogene, AP-1 transcription factor subunit |
| CA9 | Carbonic anhydrase 9 |
| TMBIM4 | Transmembrane BAX inhibitor motif containing 4 |
| PLIN2 | Perilipin 2 |
| MIR212 | microRNA 212 |
| Fer1HCH | Ferritin 1 Heavy Chain Homolog |
| AIFM2 | Apoptosis inducing factor mitochondria associated 2 |
| LAMP2 | Lysosomal associated membrane protein 2 |
| ZFP36 | ZFP36 ring finger protein |
| PROM2 | Prominin 2 |
| CHMP5 | Charged multivesicular body protein 5 |
| CHMP6 | Charged multivesicular body protein 6 |
| AKR1C1 | Aldo-keto reductase family 1 member C1 |
| CAV1 | Caveolin 1 |
| GCH1 | GTP cyclohydrolase 1 |
| HPX | hemopexin |
| LTF | lactotransferrin |
| MT2A | metallothionein 2A |
| MYC | MYC proto-oncogene, bHLH transcription factor |
| SRI | sorcin |
| ABCG2 | ATP binding cassette subfamily G member 2 |
| CAND1 | cullin associated and neddylation dissociated 1 |
| CUL1 | cullin 1 |
| CYBRD1 | cytochrome b reductase 1 |
| FBXL5 | F-box and leucine rich repeat protein 5 |
| FLVCR1 | FLVCR heme transporter 1 |
| FTL | Ferritin light chain |
| NEDD8 | NEDD8 ubiquitin like modifier |
| GLRX3 | glutaredoxin 3 |
| HEPH | hephaestin |
| FTMT | Ferritin mitochondrial |
| HMOX2 | heme oxygenase 2 |
| LCN2 | lipocalin 2 |
| MCOLN1 | mucolipin TRP cation channel 1 |
| RPS27A | ribosomal protein S27a |
| SKP1 | S-phase kinase associated protein 1 |
| SLC22A17 | solute carrier family 22 member 17 |
| SLC46A1 | solute carrier family 46 member 1 |
| STEAP2 | STEAP2 metalloreductase |
| GPX4 | Glutathione peroxidase 4 |
| TCIRG1 | T cell immune regulator 1, ATPase H+ transporting V0 subunit a3 |
| UBA52 | ubiquitin A-52 residue ribosomal protein fusion product 1 |
| UBB | ubiquitin B |
| PTGS2 | Prostaglandin-endoperoxide synthase 2 |
| DUSP1 | Dual specificity phosphatase 1 |
| NOS2 | Nitric oxide synthase 2 |
| NCF2 | Neutrophil cytosolic factor 2 |
| MT3 | Metallothionein 3 |
| ALB | Albumin |
| TXNRD1 | Thioredoxin reductase 1 |
| SRXN1 | Sulfiredoxin 1 |
| GPX2 | Glutathione peroxidase 2 |
| BNIP3 | BCL2 interacting protein 3 |
| OXSR1 | Oxidative stress responsive kinase 1 |
| SELENOS | Selenoprotein S |
| ANGPTL7 | Angiopoietin like 7 |
| HFE | homeostatic iron regulator |
| DDIT4 | DNA damage inducible transcript 4 |
| ASNS | Asparagine synthetase (glutamine-hydrolyzing) |
| TSC22D3 | TSC22 domain family member 3 |
| DDIT3 | DNA damage inducible transcript 3 |
| JDP2 | Jun dimerization protein 2 |
| SLC1A4 | Solute carrier family 1 member 4 |
| PCK2 | Phosphoenolpyruvate carboxykinase 2, mitochondrial |
| TXNIP | Thioredoxin interacting protein |
| VLDLR | Very low density lipoprotein receptor |
| GPT2 | Glutamic--pyruvic transaminase 2 |
| PSAT1 | Phosphoserine aminotransferase 1 |
| LURAP1L | Leucine rich adaptor protein 1 like |
| SLC7A5 | Solute carrier family 7 member 5 |
| HERPUD1 | Homocysteine inducible ER protein with ubiquitin like domain 1 |
| XBP1 | X-box binding protein 1 |
| HMGB1 | High mobility group box 1 |
| HMOX1 | Heme oxygenase 1 |
| ZNF419 | Zinc finger protein 419 |
| KLHL24 | Kelch like family member 24 |
| TRIB3 | Tribbles pseudokinase 3 |
| ZFP69B | ZFP69 zinc finger protein B |
| ATP6V1G2 | ATPase H+ transporting V1 subunit G2 |
| VEGFA | Vascular endothelial growth factor A |
| GDF15 | Growth differentiation factor 15 |
| TUBE1 | Tubulin epsilon 1 |
| ARRDC3 | Arrestin domain containing 3 |
| CEBPG | CCAAT enhancer binding protein gamma |
| SNORA16A | Small nucleolar RNA, H/ACA box 16A |
| RGS4 | Regulator of G protein signaling 4 |
| BLOC1S5-TXNDC5 | BLOC1S5-TXNDC5 readthrough (NMD candidate) |
| KIM-1 | Kidney injury molecule-1 |
| IL6 | Interleukin 6 |
| CXCL2 | C-X-C motif chemokine ligand 2 |
| RELA | RELA proto-oncogene, NF-kB subunit |
| HSD17B11 | Hydroxysteroid 17-beta dehydrogenase 11 |
| AGPAT3 | 1-acylglycerol-3-phosphate O-acyltransferase 3 |
| SETD1B | SET domain containing 1B, histone lysine methyltransferase |
| HSPB1 | Heat shock protein family B (small) member 1 |
| IREB2 | Iron response element binding protein 2 |
| MAPK14 | Mitogen-activated protein kinase 14 |
| MAFG | MAF bZIP transcription factor G |
| IL33 | Interleukin 33 |
| NFE2L2 | Nuclear factor, erythroid 2 like 2 |
| RPL8 | Ribosomal protein L8 |
| HAMP | Hepcidin antimicrobial peptide |
| SESN2 | Sestrin 2 |
| SLC3A2 | Solute carrier family 3 member 2 |
| DRD5 | Dopamine receptor D5 |
| DRD4 | Dopamine receptor D4 |
| MAP3K5 | Mitogen-activated protein kinase kinase kinase 5 |
| SLC2A1 | Solute carrier family 2 member 1 |
| SLC2A3 | Solute carrier family 2 member 3 |
| SLC2A6 | Solute carrier family 2 member 6 |
| SLC2A8 | Solute carrier family 2 member 8 |
| SLC2A12 | Solute carrier family 2 member 12 |
| SLC2A14 | Solute carrier family 2 member 14 |
| EIF2AK4 | Eukaryotic translation initiation factor 2 alpha kinase 4 |
| SLC40A1 | Solute carrier family 40 member 1 |
| SLC7A11 | Solute carrier family 7 member 11 |
| STEAP3 | STEAP3 metalloreductase |
| TF | Transferrin |
| TFRC | Transferrin receptor |
| UBC | Ubiquitin C |
| TFAP2C | Transcription factor AP-2 gamma |
| SP1 | Sp1 transcription factor |
| HBA1 | Hemoglobin subunit alpha 1 |
| NNMT | Nicotinamide N-methyltransferase |
| PLIN4 | Perilipin 4 |
| HIC1 | HIC ZBTB transcriptional repressor 1 |
| STMN1 | Stathmin 1 |
| RRM2 | Ribonucleotide reductase regulatory subunit M2 |
| CAPG | Capping actin protein, gelsolin like |
| HNF4A | Hepatocyte nuclear factor 4 alpha |
| NGB | Neuroglobin |
| YWHAE | Tyrosine 3-monooxygenase/tryptophan 5-monooxygenase activation protein epsilon |
| GABPB1 | GA binding protein transcription factor subunit beta 1 |
| AURKA | Aurora kinase A |
| MIR4715 | microRNA 4715 |
| RIPK1 | Receptor interacting serine/threonine kinase 1 |
| PRDX1 | Peroxiredoxin 1 |
| MIR30B | microRNA 30b |
