# Supplementary figures and images for: Integrated clinical characteristics and omics analysis identifies a ferroptosis and iron-metabolism-related lncRNA signature for predicting prognosis and therapeutic responses in ovarian cancer
Source: J Ovarian Res. 2022 Jan 20;15:10. doi: 10.1186/s13048-022-00944-y (PMC8772079; doi:10.1186/s13048-022-00944-y)

a

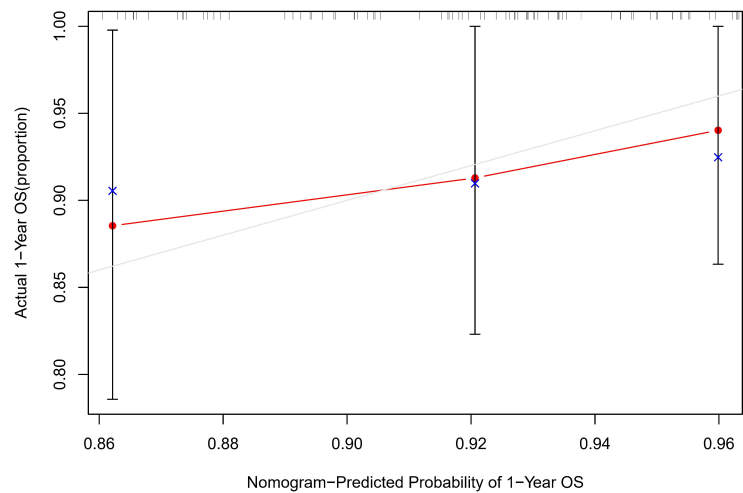

b

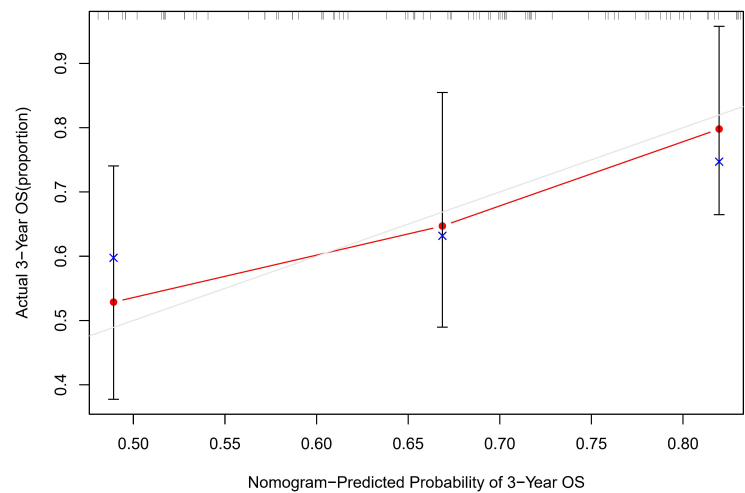

c

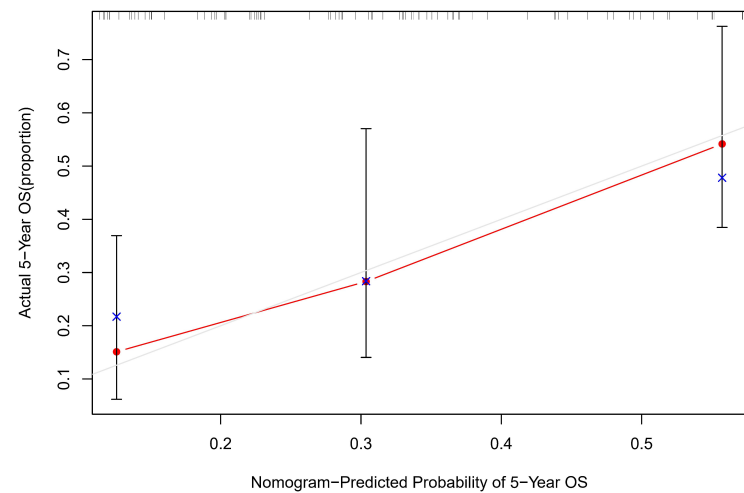

d

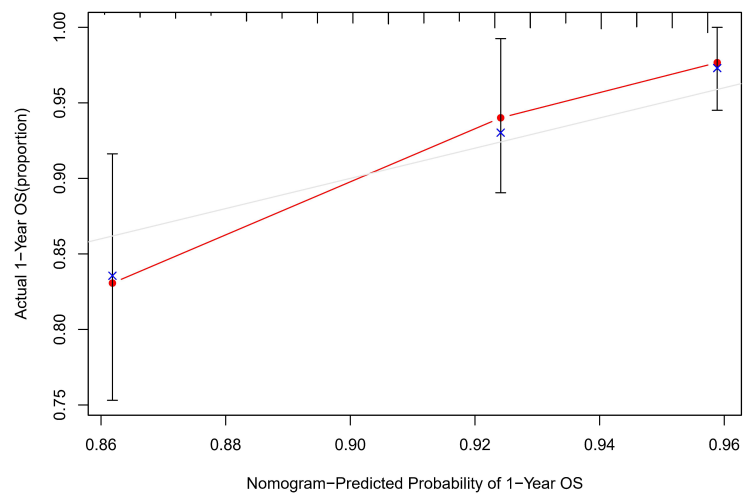

e

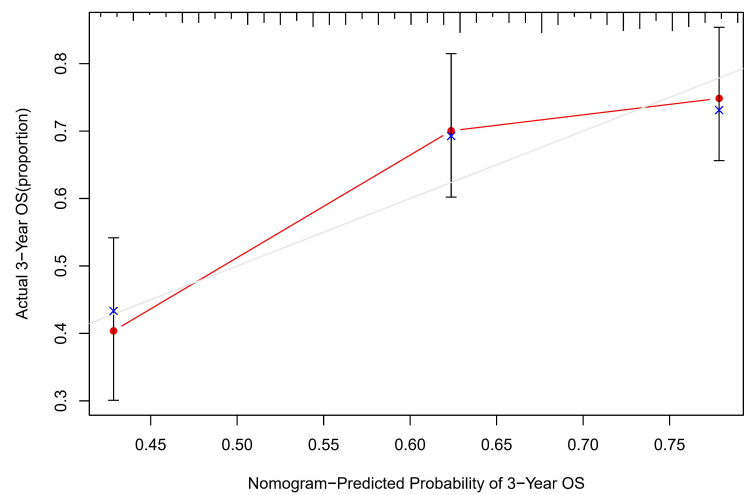

f

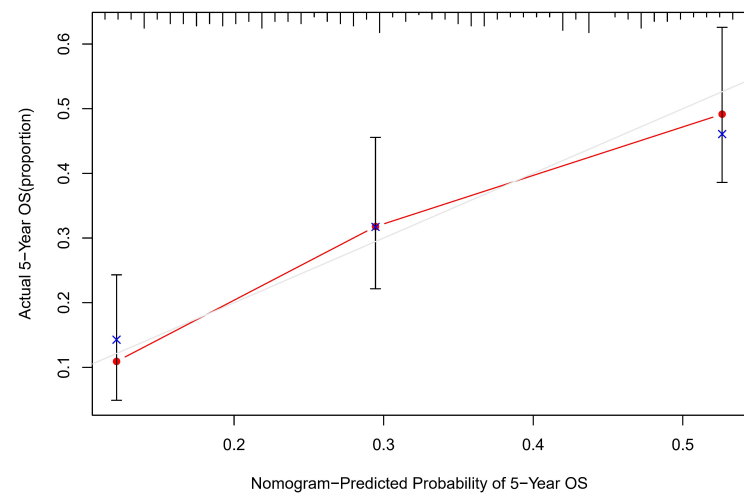

Supplement: Supplementary file 2 — Additional file 2. Figure S1. Calibration curve of nomogram. (a-b) Calibration curve of nomogram based on FIRLs signature for OS prediction at 1 year (a), 3 year (b) , and 5 year (c) in the training set; (d-f) Calibration curve of nomogram based on FIRLs signature for OS prediction at 1 year (d), 3 year (e), and 5 year (f) in the testing set. [file 13048_2022_944_MOESM2_ESM.pdf]

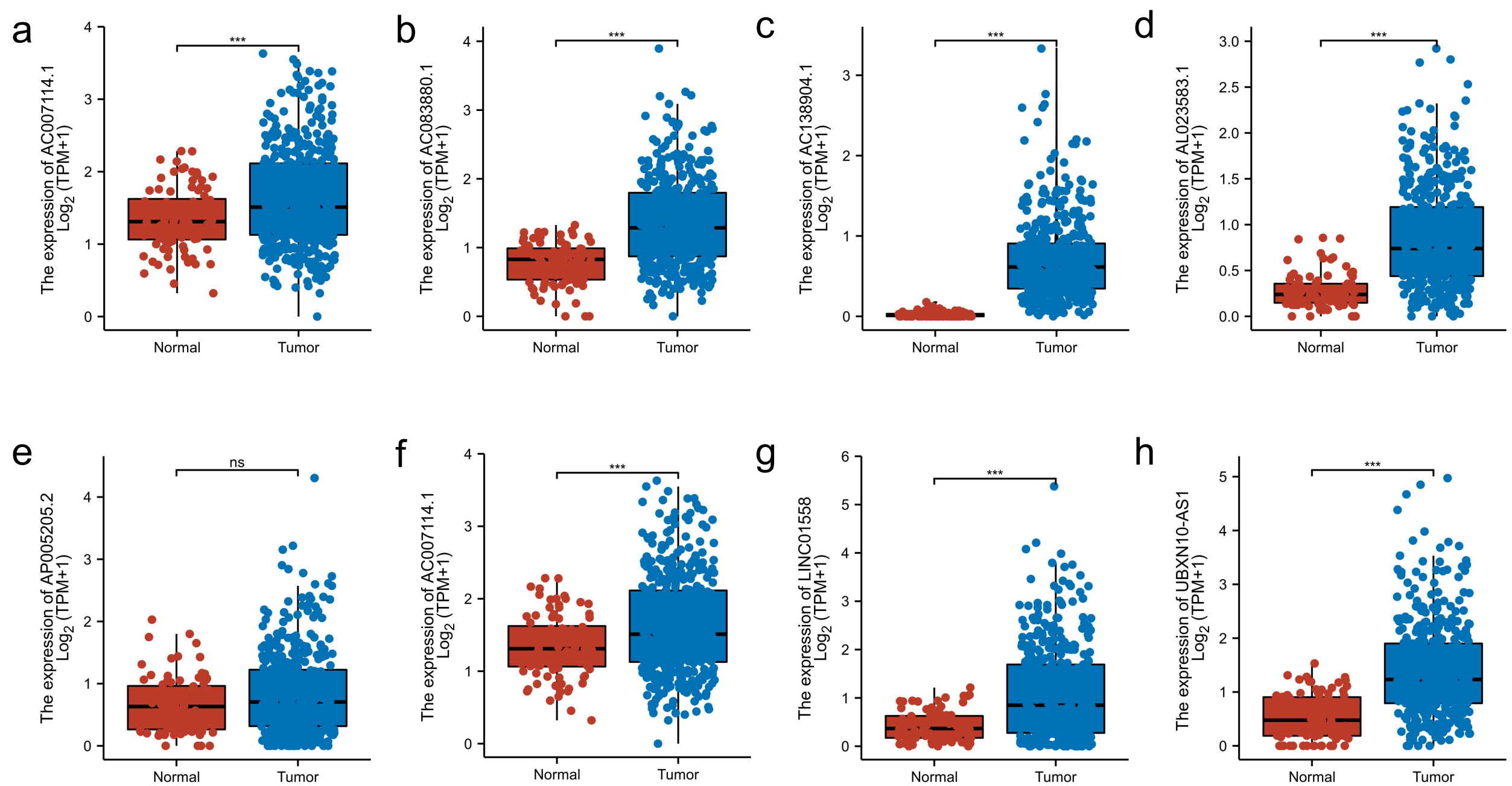

Supplement: Supplementary file 3 — Additional file 3. Figure S2. The expression of 8-FIRLs in normal and OC samples. (a) The expression of AC138904.1; (b)The expression of AP005205.2; (c) The expression of AC007114.1; (d) The expression of LINC00665; (e) The expression of UBXN10-AS1; (f) The expression of AC083880.1; (g) The expression of LINC01558; (h) The expression of AL023583.1; *P < 0.05; **P < 0.01; ***P < 0.001. [file 13048_2022_944_MOESM3_ESM.pdf]
